# A clay-shoveler's fracture with renal transplantation and osteoporosis: a case report

**DOI:** 10.1186/1752-1947-2-187

**Published:** 2008-06-02

**Authors:** Koray Unay, Omer Karatoprak, Nadir Sener, Korhan Ozkan

**Affiliations:** 1 Department of Orthopedics and Traumatology, Goztepe Research and Training Hospital, Istanbul, Turkey; 2 Department of Orthopedics and Traumatology, Kadikoy Florence Nightingale Hospital, Istanbul, Turkey; 3 Department of Orthopedics and Traumatology, Bursa Acibadem Hospital, Bursa, Turkey

## Abstract

**Introduction:**

Clay-shoveler's fracture is a rare cervicodorsal spinous process fracture and there is little information regarding the prognosis of patients with this condition in conjunction with osteoporosis and corticosteroid use.

**Case presentation:**

A 39-year-old man was admitted to our institution with a 6-month history of cervicodorsal pain prior to admission. The patient had previously undergone renal transplantation and was on corticosteroids, and had developed osteoporosis. We treated him with a cervical collar, non-steroidal anti-inflammatory agents and alendronate. The patient was advised against performing weight-bearing activities for 6 months.

**Conclusion:**

Clay-shoveler's fracture with osteoporosis and corticosteroid use presented by fracture of the cervicodorsal aspect of the spinous processes may be successfully treated with a collar, alendronate and long-term rest.

## Introduction

Clay-shoveler's fracture is a rare condition that was first observed at the beginning of the 20th century in non-industrialized areas, particularly in weight-bearing workers. Such fractures are rarely observed in present times with the advent of industrialization and reduced weight-bearing activities. Clay-shoveler's fracture is a result of the shear forces exerted particularly by the trapezius and rhomboid muscles on the upper and lower spinous processes of the cervical and dorsal vertebrae, respectively. Typically, the patient complains of an acute, burning, knife-like pain at the cervicodorsal site. Such fractures generally respond to treatment. However, there are no data regarding the duration of treatment or follow-up of patients with concurrent osteoporosis [1,2].

In this case report, we discuss the treatment process and follow-up of Clay-shoveler's fracture diagnosed late in a bellboy with osteoporosis who had undergone renal transplantation and was being treated with corticosteroids.

## Case presentation

We report the case of a 39-year-old man with a height of 178 cm and weight of 72 kg, working as a bellboy in a hotel. He had developed renal insufficiency 8 years previous, had undergone hemodialysis and had received a left renal transplantation after 2.5 years of hemodialysis. The patient had taken 8 mg methylprednisolone once a day for 3.5 years following the renal transplantation. The nephrologist following-up our patient decreased the methylprednisolone dose to 4 mg 1 week before admission.

The patient had complained of increasing back and neck pain for 6 months prior to admission. He had been diagnosed with paravertebral spasm at two different locations. There was no history of trauma. After admission, the patient showed no improvement, and experienced worsening pain. The pain was an acute shooting pain resembling an electrical flash at the cervicodorsal site. Weight bearing was an occupational requirement, and the patient claimed that there was more pain while bearing heavy weights. There was also a history of night pains at the cervicodorsal site and pain and paresthesia of the upper extremity and chest.

**Figure 1:**
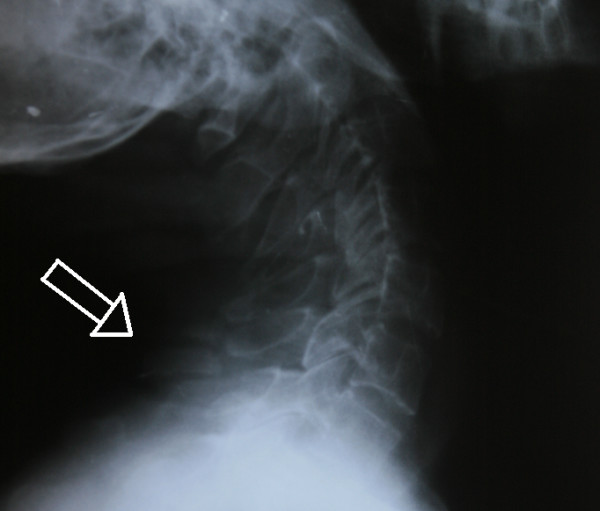
**X-ray of the cervicodorsal site, lateral view**. White arrow shows fractured spinous processes.

**Figure 2:**
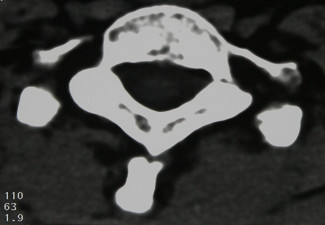
Computerized tomography scans showing axial section of the first dorsal vertebra.

Neurological examination was normal. There was pain at the cervicodorsal site during neck movements, particularly during neck flexion. Based on the suspicious images of the spinous processes of the C7 and D1 vertebrae in the X-rays of the cervical and dorsal vertebrae (Figure 1), cervicodorsal computerized tomography was performed (Figure 2). The spinous process fractures of the C7, D1 and D2 vertebrae were diagnosed as Clay-shoveler's fractures. The patient was treated with a cervical collar for 1.5 months. Due to the patient's history of renal transplantation and corticosteroid use, whole body bone densitometry was performed. The mean lumbar spine T-score was -2.7, and the mean femoral neck T-score was -2.1. The patient was given 10 mg oral alendronate once a day. At the second month of follow-up, the control dual energy X-ray absorptiometry (DEXA) value of the mean lumbar spine T-score regressed to -2.6 and the mean femoral neck T-score regressed to -2.0, and at the fourth month, the scores had regressed to -2.4 and -1.9. Following the collar and alendronate treatment, the patient's pain regressed rapidly in 1.5 months. The patient was followed-up for 12 months, and he continued on oral methylprednisolone 4 mg/day during the follow-up.

**Figure 3:**
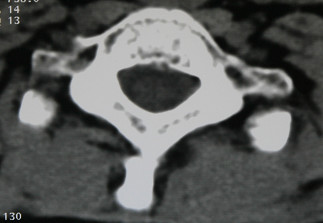
Control computerized tomography scan at 12 months showing axial section of the first dorsal vertebra.

Alendronate treatment was discontinued when the patient's T-scores reached -2.0 (the mean lumbar spine T-score) and -1.3 (the mean femoral neck T-score) on the 12-month DEXA scan. For evaluation of the patient's renal function, urinary creatinine clearance was measured at 1, 2, 3, 6, 9, and 12 months after the alendronate treatment. No renal malfunction was observed. He was given a rest report that prohibited him from weight-bearing activities for a total of 6 months. Control computerized tomography scanning was performed 6 months after the resolution of the pain (Figure 3) and the fracture sites were evaluated for union of the fractures. There was no pain at the follow-up examination 24 months after the fracture.

## Discussion

Clay-shoveler's fracture may occur through direct trauma on the flexed spine or through shear forces; shear forces seem to have been the cause in this case. Cases of Clay-shoveler's fracture have been reported in the literature; however, these cases were in otherwise healthy individuals with no history of prior disease. We present the case of a bellboy with a history of renal transplantation, long-term corticosteroid use and osteoporosis. In such patients, it is important to be cautious with regard to renal function. Thus, while following-up our patient during alendronate treatment, we also followed-up for creatinine clearance [3,4]. He was unable to change his job and thus needed to continue his weight-bearing activities. Rest was discontinued on the complete resolution of the pain 12 months after the fracture, when the patient's response to light weight-bearing exercises improved. Hence, we ensured complete healing by advising long-term rest. The patient was followed-up for 12 months after resuming work, and he experienced no further symptoms. This case indicates that it is not possible for a patient with osteoporosis and Clay-shoveler's fracture to bear weight within 12 months of the fracture.

These injuries are known to be stable but painful. In most patients, immobilization of the neck with a cervical collar and restriction of physical activity for 4 to 6 weeks frequently result in pain relief, but healing of the fractures does not usually occur [5,6]. Our patient experienced a decrease in pain with treatment, which consisted of wearing a cervical collar, medication with alendronate and weight-bearing restrictions beginning on admission 6 months after the fracture. During the 1.5 months of collar treatment, the patient reported that his pain increased rapidly on occasions when he did not use the collar. The restriction of neck flexion by the collar was an important factor in decreasing the pain, by reducing the tension of the posterior elements at the cervicodorsal site.

## Conclusion

This case demonstrated a specific painful site and a relevant occupational history; it is important not to overlook such factors in a clinical setting. Our patient's history included osteoporosis, renal transplantation and corticosteroid treatment; he earned and continues to earn his livelihood by bearing weight, and presented with Clay-shoveler's fracture by fracture of the cervicodorsal aspect of the spinous processes. This was successfully treated with a cervical collar, alendronate and long-term rest, to the extent that the patient was able to resume weight-bearing activities approximately 12 months after the fracture. There was no pain at the follow-up examination 24 months after the fracture.

## Abbreviations

DEXA: dual energy X-ray absorptiometry.

## Competing interests

The authors declare that they have no competing interests.

## Consent

Written informed consent was obtained from the patient for publication of this case report and accompanying images. A copy of the written consent is available for review by the Editor-in-Chief of this journal.

## Authors' contributions

KU contributed to conception and design of the report, and carried out the literature search, manuscript preparation and manuscript review, OK and NS were involved in the literature review and helped draft part of the manuscript, KO contributed to conception and design of the report. All authors read and approved the final manuscript.
